# Association of baseline Life’s Essential 8 score and trajectories with carotid intima-media thickness

**DOI:** 10.3389/fendo.2023.1186880

**Published:** 2023-06-02

**Authors:** Qian Liu, Haozhe Cui, Shuohua Chen, Dongyan Zhang, Wei Huang, Shouling Wu

**Affiliations:** ^1^Lanzhou University Second Hospital, Lanzhou University, Lanzhou, China; ^2^Department of Cardiology, Kailuan General Hospital, Tangshan, China; ^3^School of Medicine, Nankai University, Tianjin, China; ^4^Ultrasound Medicine Department, Kailuan General Hospital, Tangshan, China

**Keywords:** Life’s Essential 8, cardiovascular health, carotid intima-media thickness, trajectories, prospective cohort study

## Abstract

**Objective:**

We aimed to examine the association between the baseline Life’s Essential 8 (LE8) score and LE8 score trajectories with the continuous carotid intima-media thickness (cIMT) as well as the risk of high cIMT.

**Methods:**

The Kailuan study has been an ongoing prospective cohort study since 2006. A total of 12,980 participants who completed the first physical examination and cIMT detection at follow-up without a history of CVD and missing data on the component of LE8 metrics in or before 2006 were finally included in the analysis. The LE8 score trajectories were developed from 2006 to 2010 using trajectory modeling of the SAS procedure Proc Traj. The measurement and result review of the cIMT were performed by specialized sonographers using standardized methods. According to quintiles of baseline LE8 score, participants were categorized into five groups: *Q*1, *Q*2, *Q*3, *Q*4, and *Q*5. Similarly, based on their LE8 score trajectories, they were classified into four groups: very low-stable group, low-stable group, median-stable group, and high-stable group. In addition to continuous cIMT measurement, we determined the high cIMT based on the age (by 5 years) and sex-specific 90th percentile cut point. To address aims 1 and 2, the association between baseline/trajectory groups and continuous cIMT/high cIMT was assessed by using SAS proc genmod to calculate β, relative risk (RR), and 95% confidence intervals (CI).

**Results:**

A total of 12,980 participants were finally included in aim 1, and 8,758 participants met aim 2 of the association between LE8 trajectories and cIMT/high cIMT. Compared with the *Q*1 group, the continuous cIMT for *Q*2, *Q*3, *Q*4, and *Q*5 groups were thinner; the other groups had a lower risk of high cIMT. For aim 2, the results indicated that compared with a very low-stable group, the cIMT for the low-stable group, the median-stable group, and the high-stable group were thinner (−0.07 mm [95% CI −0.10~0.04 mm], −0.10 mm [95% CI −0.13~−0.07 mm], −0.12 mm [95% CI −0.16~−0.09 mm]) and had a lower risk of high cIMT. The RR (95% CI) for high cIMT was 0.84 (0.75~0.93) in the low-stable group, 0.63 (0.57~0.70) in the median-stable group, 0.52 (0.45~0.59) in the high-stable group.

**Conclusions:**

In summary, our study revealed that high baseline LE8 scores and LE8 score trajectories were associated with lower continuous cIMT and attenuated risk of high cIMT.

## Introduction

In 2010, the American Heart Association (AHA) set an ideal cardiovascular health (CVH) goal to improve the CVH of all Americans by 20% while reducing mortality from cardiovascular disease (CVD) by 20% over the next decade (1). In 2022, AHA updated and strengthened the concept of CVH to encourage individuals and populations to further improve CVH and proposed a new method for defining and quantifying CVH metrics known as “Life’s Essential 8” (LE8). In this method, sleep health was added as a new component of CVH (2).

Poor baseline CVH scores and low CVH score trajectories, defined by the old Life’s Simple 7 (LS7), have previously been associated with increased burden from hypertension, CVD, and subclinical atherosclerosis (3–5). Carotid intima-media thickness (cIMT) measured by carotid ultrasonography has been shown to more accurately identify populations at high risk of CVD than using major risk factors (6). More evidence has further supported an association between CVH and cIMT, whether baseline CVH or CVH trajectories that were defined by previous LS7 metrics (7, 8). However, the association between baseline LE8 and cIMT has not yet been studied. Furthermore, several previous studies have found that CVH components may be influenced by many factors and change over time. As a result, describing the longitudinal pattern of the CVH score using CVH score trajectories provides a more reliable result.

As a result, we used data from the Kailuan study to investigate the association between baseline CVH scores defined by new LE8 metrics and cIMT, and we further explored the association between new LE8 score trajectories and cIMT.

## Methods

### Study population and design

The Kailuan study is an ongoing prospective cohort study conducted among a functional community population in Tangshan, China, since 2006. All participants are followed up every 2 years, with a total of six follow-ups completed so far. The details of the Kailuan study have been described previously (9, 10). The participants were randomly chosen from the participants who were enrolled in the first physical examinations, and they completed the assessment of cIMT by ultrasonography from the third to seventh physical examinations (11).

Briefly, 13,934 participants completed the first physical examination and cIMT detection at follow-up. We excluded 954 participants who could meet the following criteria: (1) 222 participants with a history of CVD; and (2) 732 participants with missing data on the component of LE8 metrics. Therefore, a total of 12,980 participants were finally included in the analysis.

**Figure 1:**
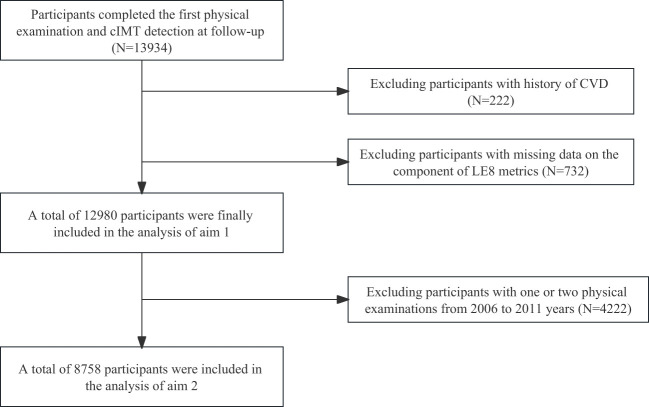
Flow chart of the current study.

**Figure 2:**
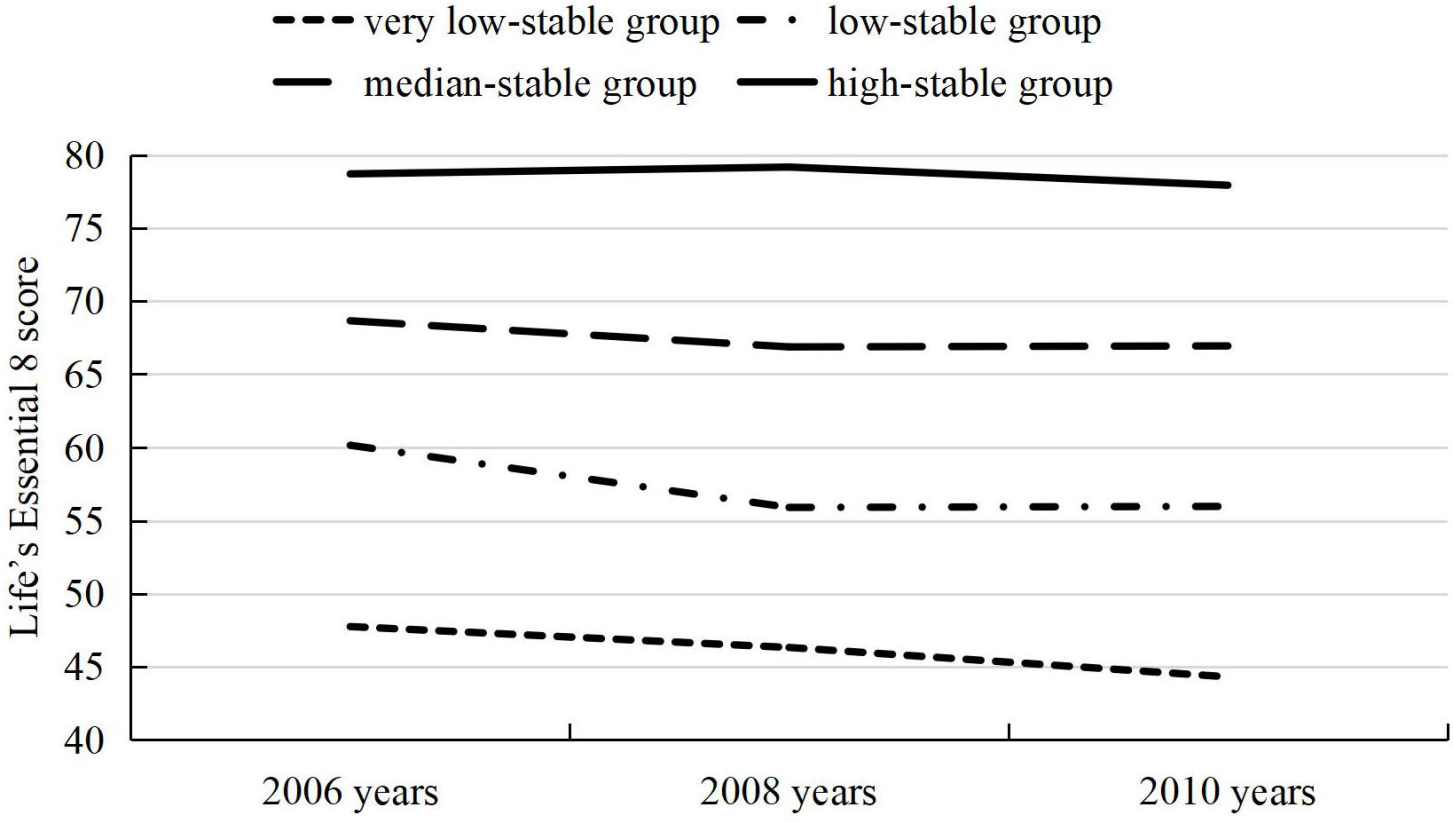
Life’s Essential 8 trajectories.

Aim 1 of this study was to investigate the associations between baseline LE8 score and continuous cIMT/high cIMT. Aim 2 of this study was to investigate the association between LE8 trajectories and continuous cIMT/high cIMT. In addition, the LE8 score trajectories were developed from the first physical examinations to the third physical examinations (8,758 participants met the criteria). The participants were divided into four trajectory patterns according to LE8 scores (very low-stable group, low-stable group, median-stable group, and high-stable group). **Figure 1** shows the flow chart of inclusion and exclusion criteria. **Figure 2** shows the four trajectory groups in the present study.

The Declaration of Helsinki was followed in this study. This study was approved by the ethics committee of Kailuan General Hospital. All participants signed an informed consent form.

### LE8 metrics

Information on demographic characteristics, medical history, and lifestyle factors, including tobacco exposure, physical activity, salt intake, sleep duration, drinking status, education status, marital status, income status, and so on, was collected by trained staff through standardized questionnaires, as described previously (12).

After participants had fasted for at least 8 h, 5 ml of blood was drawn to measure biochemical indexes such as total cholesterol (TC), low-density lipoprotein cholesterol (LDL-C), high-density lipoprotein cholesterol (HDL-C), high-sensitive C-reactive protein (Hs-CRP), and fasting blood glucose (FBG). All biochemical analyses were performed using an auto-analyzer (Hitachi 747; Hitachi, Tokyo, Japan). non-HDL-C = TC − HDL-C. Blood pressure was measured on the day of the physical examination between 7:00 and 9:00 a.m., without smoking or tea/coffee consumption allowed 30 min before measurement, and after participants had remained in a seated position for at least 5 min. The measurement was repeated three times with an interval of 1–2 min, and the mean value was taken. Blood pressure and blood samples were measured by trained staff through standardized methods, as described previously (13). Body mass index (BMI) was calculated as weight (kg)/height (m)^2^.

### Diet

Owing to the lack of detailed dietary data and taking into account the influence of consumption of salt intake, tea, and high-fat foods on CVD risks among the Chinese population, salt intake, tea consumption, and high-fat foods intake based on a questionnaire were used as a surrogate measure for diet quality, as previously described (14–17).

### Fasting blood glucose

Owing to the lack of hemoglobin A1c and the close relationship between hemoglobin A1c and mean FBG, A1C was replaced by FBG as blood glucose metrics. FBG (mg/dl) = 28.7 * A1C − 46.7 (18).

According to the updated and enhanced approach to assess CVH, definition and scoring for the components of LE8, including four health behaviors (diet, physical activity, smoking status, and sleep health) and four health factors (BMI, non-HDL-C, FBG, and blood pressure), were shown in **Supplementary Table S1** (**Additional file 1**) (2). Each component of LE8 was scored on a scale of 0 to 100 points. The overall LE8 score was calculated by summing the scores for each of the eight metrics together and dividing the total by 8, to provide a LE8 score ranging from 0 to 100. Participants were categorized into five groups (*Q*1 [<57.29], *Q*2 [57.29~64.79], *Q*3 [64.79~69.79], *Q*4 [69.79~76.25], and *Q*5 [≥76.25]) according to quintiles of LE8 score, and the lowest quintile (*Q*1) was used as the reference group.

### Covariates

In recent years, alcohol consumption was defined as the daily consumption of at least 100 ml of beverages (or liquors) containing alcohol of ≥ 50% alcohol/day (19). Education status was categorized as high school or above and junior high school or below. Marital status was categorized as in married vs. not married. Income status was categorized into ≤ 1,000 and > 1,000 RMB/month (20).

### cIMT

The cIMT was measured using a *Philips HD*-15 color ultrasonic diagnostic instrument (with a probe frequency of 5–12 MHz). The participants assumed the supine position, and the cIMT of the bilateral carotid arteries, including the common carotid artery, carotid bifurcation, and internal and external carotid arteries, were measured. Two professional physicians completed the measure: one operated on and another recorded. The examination results were then reviewed by two independent operators, and discrepancies between their evaluations were resolved by consensus. The mean of the maximum cIMT readings of three right and three left far walls for common, bifurcation, and internal segments were used. The standardized methods have been previously described (21). In this study, the definition and measurements of cIMT followed the Mannheim Carotid Intima-Media Thickness and Plaque Consensus (2004–2006–2011) (22). In addition to the continuous cIMT measurement, an age- (5 years) and sex-specific 90th percentile cut point determined high cIMT (23). High cIMT was defined as a cIMT value equal to or greater than the given cut point. The cut points for current analyses are shown in **Supplementary Table S2** (**Additional file 1**).

### Statistical analysis

Continuous, normally distributed variables were presented as the mean ± standard deviation (SD), and groups were compared using one-way ANOVA. Category variables were presented by number and percentage (%), with comparisons between groups by Chi-square test. In the primary analysis for aim 2, participants with LE8 scores in 2006, 2008, and 2010 were included in trajectory modeling using the SAS procedure Proc Traj. The best model fit was assessed using the Bayesian information criterion and the number of participants in each trajectory (> 5% of the overall population) (13, 24). Finally, the participants were divided into four trajectory patterns according to both baseline LE8 scores and LE8 scores over time (very low-stable group, low-stable group, median-stable group, and high-stable group).

To address aims 1 and 2, the association between baseline/trajectory groups and continuous cIMT/high cIMT was assessed by using SAS proc genmod to calculate β, relative risk (RR), and 95% confidence intervals (CI). Models were sequentially adjusted for age, sex, current drinker, education status, income status, and marital status. Models of association between trajectory groups and continuous cIMT/high cIMT were further adjusted for either baseline or proximal LE8 score because of the correlation between baseline and proximal scores.

To verify the robustness of the results, several sensitivity analyses were performed (male participants, participants without hypertension, participants without diabetes, participants without lipid-lowering drugs/antihypertensive drugs/hypoglycemic drugs, and participants in junior high school or below).

All statistical analyses were conducted using SAS 9.4 (SAS Institute, Inc., Cary, NC, USA), and a two-tailed *p* < 0.05 was considered statistically significant.

## Results

### Baseline characteristics

Among 12,980 participants examined at baseline, 8,203 (63.20%) were men, and the mean age of the study participants was (48.41 ± 11.63) years. The baseline characteristics of participants according to the quintiles of LE8 score are presented in **Table 1**. The baseline characteristics of participants according to sex are presented in **Supplementary Table S3** (**Additional file 1**). There is an increasing trend of the percentage with high school or above and greater than 1,000 RMB/month and a decreasing trend of the age, cIMT, and percentage with current drinkers from the *Q*1 to *Q*5 group. Analysis of aim 2 included 8,758 participants who met the inclusion criteria. We identified four different trajectories, including high-stable group (1,903 [21.73%]), median-stable group (3,249 [37.10%]), low-stable group (2,944 [33.61%]), and very low-stable group (662 [7.56%]). There is an increasing trend in the percentage of high school or above and a decreasing trend in the percentage of current drinkers from the very low-stable group to the high-stable group [**Supplementary Table S4** (**Additional file 1**)].

**Table 1:** Baseline characteristics of participants according to the quintiles of LE8 score in 2006.

Characteristic	Quintile of LE8 score					*p*-value
	*Q*1 (<57.29)	*Q*2 (57.29~64.79)	*Q*3 (64.79~69.79)	*Q*4 (69.79~76.25)	*Q*5 (≥76.25)	
Number of participants	2,555	2,536	2,277	2,992	2,620	
Age (years)	50.51 ± 10.19	51.29 ± 10.96	50.19 ± 10.98	47.76 ± 11.77	42.76 ± 11.94	<0.01
Male (*n* (%))	2,272 (88.92)	1,989 (78.43)	1,595 (70.05)	1,690 (56.48)	657 (25.08)	<0.01
BMI (kg/m^2^)	27.01 ± 3.35	26.10 ± 3.39	25.45 ± 3.03	24.37 ± 2.88	22.21 ± 2.48	<0.01
Systolic blood pressure (mmHg)	141.85 ± 19.68	137.42 ± 19.70	132.59 ± 18.01	125.82 ± 16.29	109.99 ± 12.17	<0.01
Diastolic blood pressure (mmHg)	90.46 ± 11.45	87.08 ± 11.30	85.02 ± 10.54	81.27 ± 9.42	71.81 ± 7.29	<0.01
FBG (mmol/L)	6.23 ± 2.22	5.60 ± 1.61	5.29 ± 1.11	5.01 ± 0.81	4.86 ± 0.57	<0.01
HDL-C (mmol/L)	1.56 ± 0.38	1.58 ± 0.40	1.59 ± 0.36	1.61 ± 0.36	1.59 ± 0.35	<0.01
TC (mmol/L)	5.32 ± 1.58	4.94 ± 1.45	4.76 ± 1.23	4.52 ± 1.11	4.43 ± 0.90	<0.01
non-HDL-C (mmol/L)	3.77 ± 1.57	3.36 ± 1.45	3.16 ± 1.24	2.91 ± 1.11	2.84 ± 0.87	<0.01
Sleep duration (h)	6.77 ± 2.88	7.24 ± 2.41	7.53 ± 2.50	7.66 ± 2.17	7.63 ± 1.68	<0.01
cIMT (mm)	0.95 ± 0.45	0.93 ± 0.47	0.89 ± 0.32	0.85 ± 0.19	0.79 ± 0.18	<0.01
Hs-CRP (<3 mg/L; *n* (%))	2,117 (82.86)	2,147 (84.66)	1,961 (86.12)	2,584 (86.36)	2,294 (87.56)	<0.01
Marital status (in married; *n* (%))	2,464 (96.44)	2,444 (96.41)	2,228 (97.85)	2,903 (97.03)	2,487 (94.92)	<0.01
High school or above (*n* (%))	539 (21.10)	514 (20.28)	405 (17.79)	613 (20.49)	1,171 (44.69)	<0.01
Income status (>1,000 RMB/month; *n* (%))	228 (8.93)	208 (8.21)	138 (6.07)	182 (6.08)	275 (10.50)	<0.01
Current drinker (*n* (%))	1,542 (60.35)	963 (37.97)	498 (21.87)	481 (16.08)	425 (16.22)	<0.01
Smoking status (*n* (%))						
Never	681 (26.65)	1,487 (58.64)	1,820 (79.93)	2,716 (90.78)	2,551 (97.37)	<0.01
Former smokers quit ≥1 year	154 (6.03)	173 (6.82)	100 (4.39)	80 (2.67)	34 (1.30)	
Current <1 cigarette/day	109 (4.27)	101 (3.98)	54 (2.37)	34 (1.14)	10 (0.38)	
Current ≥1 cigarette/day	1,611 (63.05)	775 (30.56)	303 (13.31)	162 (5.41)	25 (0.95)	
Physical activity (*n* (%))						
Never	571 (22.35)	192 (7.57)	75 (3.29)	84 (2.81)	52 (1.98)	<0.01
1–3 times/week	1,699 (66.50)	1,932 (76.18)	1,867 (81.99)	2,561 (85.59)	2,130 (81.30)	
≥4 times/week	285 (11.15)	412 (16.25)	335 (14.71)	347 (11.60)	438 (16.72)	
Sodium intake (*n* (%))						
<6 g/day	227 (8.88)	218 (8.60)	172 (7.55)	212 (7.09)	358 (13.66)	<0.01
6–10 g/day	1,783 (69.78)	2,031 (80.09)	1,956 (85.90)	2,642 (88.30)	2,112 (80.61)	
>10 g/day	545 (21.33)	287 (11.32)	149 (6.54)	138 (4.61)	150 (5.73)	
High-fat food intake (*n* (%))						
<1 time/week	187 (7.32)	188 (7.41)	145 (6.37)	184 (6.15)	314 (11.98)	<0.01
1–3 times/week	1,941 (75.97)	2,096 (82.65)	2,019 (88.67)	2,690 (89.91)	2,192 (83.66)	
>3 times/week	427 (16.71)	252 (9.94)	113 (4.96)	118 (3.94)	114 (4.35)	
Tea intake (*n* (%))						
Never	1,779 (69.63)	1,885 (74.33)	1,882 (82.65)	2,519 (84.19)	1,836 (70.08)	<0.01
<1 time/month	139 (5.44)	95 (3.75)	64 (2.81)	93 (3.11)	210 (8.02)	
1–3 times/month	181 (7.08)	164 (6.47)	102 (4.48)	109 (3.64)	214 (8.17)	
1–3 times/week	158 (6.18)	114 (4.50)	88 (3.86)	115 (3.94)	158 (6.03)	
≥4 times/week	298 (11.66)	278 (10.96)	141 (6.19)	156 (5.21)	202 (7.71)	
Lipid-lowering drugs (*n* (%))	46 (1.98)	29 (1.23)	22 (1.01)	9 (0.31)	5 (0.20)	<0.01
Antihypertensive drugs (*n* (%))	570 (22.98)	367 (14.76)	173 (7.72)	115 (3.87)	40 (1.53)	<0.01
Diabetes history (*n* (%))	553 (21.64)	304 (11.99)	127 (5.58)	55 (1.84)	12 (0.46)	<0.01

LE8, Life’s Essential 8; BMI, body mass index; TC, total cholesterol; LDL-C, low-density lipoprotein cholesterol; HDL-C, high-density lipoprotein cholesterol; non-HDL-C, non-high-density lipoprotein cholesterol; Hs-CRP, high-sensitive C-reactive protein; FBG, fasting blood glucose; cIMT, carotid intima-media thickness.

### Association between baseline LE8 score with continuous cIMT/high cIMT

The quintiles of LE8 score were associated with continuous cIMT and high cIMT (**Table 2**). After adjustment for age, sex, current drinker, education status, income status, and marital status, compared with *Q*1 group, the cIMT for *Q*2, *Q*3, *Q*4, and *Q*5 groups were thinner (−0.02 mm [95% CI −0.04~−0.01 mm], −0.05 mm [95% CI −0.07~−0.03 mm], −0.07 mm [95% CI −0.08~−0.05 mm], −0.07 mm [95% CI −0.09~−0.05 mm]). Compared with the *Q*1 group, the other groups had a lower risk of high cIMT; the RR (95% CI) for high cIMT was 0.68 (0.59~0.79) in the *Q*2 group, 0.61 (0.53~0.71) in the *Q*3 group, 0.65 (0.56~0.74) in the *Q*4 group, and 0.51 (0.43~0.60) in the *Q*5 group.

**Table 2:** Association between baseline LE8 score with continuous cIMT/high cIMT.

	Adjusted^a^			Adjusted^b^		
	*β* (95% CI)	RR (95% CI)	*p*-value	*β* (95% CI)	RR (95% CI)	*p*-value
cIMT (mm)						
*Q*1	Reference			Reference		
*Q*2	−0.02 (−0.04~0.00)		0.05	−0.02 (−0.04~−0.01)		0.02
*Q*3	−0.05 (−0.06~−0.03)		<0.01	−0.05 (−0.07~−0.03)		<0.01
*Q*4	−0.06 (−0.08~−0.04)		<0.01	−0.07 (−0.08~−0.05)		<0.01
*Q*5	−0.07 (−0.09~−0.05)		<0.01	−0.07 (−0.09~−0.05)		<0.01
Per 10-point increase	−0.02 (−0.03~−0.02)		<0.01	−0.03 (−0.03~−0.02)		<0.01
High cIMT						
Q1		Reference			Reference	
Q2		0.71 (0.62~0.81)	<0.01		0.68 (0.59~0.79)	<0.01
Q3		0.65 (0.56~0.76)	<0.01		0.61 (0.53~0.71)	<0.01
Q4		0.69 (0.60~0.79)	<0.01		0.65 (0.56~0.74)	<0.01
Q5		0.55 (0.47~0.64)	<0.01		0.51 (0.43~0.60)	<0.01
Per 10-point increase		0.83 (0.79~0.87)	<0.01		0.81 (0.77~0.84)	<0.01

a Adjusted for age and sex.

b Adjusted for age, sex, current drinker, education status, income status, and marital status.

### Association between LE8 score trajectories with continuous cIMT/high cIMT

The LE8 score trajectories were also associated with continuous cIMT and high cIMT (**Table 3**). After adjustment for age, sex, current drinker, education status, income status, and marital status, compared with a very low-stable group, the cIMT for a low-stable group, median-stable group, and high-stable group were thinner (−0.07 mm [95% CI −0.10~−0.04 mm], −0.10 mm [95% CI −0.13~−0.07 mm], −0.12 mm [95% CI −0.16~−0.09mm]). Compared with the very low-stable group, the other groups had a lower risk of high cIMT; the RR (95% CI) for high cIMT was 0.84 (0.75~0.93) in the low-stable group, 0.63 (0.57~0.70) in the median-stable group, and 0.52 (0.45~0.59) in the high-stable group. These patterns remained consistent even after adjustment for the baseline or proximal LE8 score.

**Table 3:** Association between LE8 score trajectory groups with continuous cIMT/high cIMT.

	Adjusted^a^			Adjusted and baseline score^b^			Adjusted and proximal score^c^		
	*β* (95% CI)	RR (95% CI)	*p*-value	*β* (95% CI)	RR (95% CI)	*p*-value	*β* (95% CI)	RR (95% CI)	*p*-value
cIMT (mm)									
Very low-stable group	Reference								
Low-stable group	−0.07 (−0.10~−0.04)		<0.01	−0.05 (−0.08~−0.01)		<0.01	−0.06 (−0.09~−0.04)		<0.01
Median-stable group	−0.10 (−0.13~−0.07)		<0.01	−0.06 (−0.10~−0.03)		<0.01	−0.09 (−0.13~−0.06)		<0.01
High-stable group	−0.12 (−0.16~−0.09)		<0.01	−0.07 (−0.11~−0.02)		<0.01	−0.11 (−0.15~−0.07)		<0.01
High cIMT									
Very low-stable group		Reference			Reference			Reference	
Low-stable group		0.84 (0.75~0.93)	<0.01		0.89 (0.79~0.99)	0.04		0.88 (0.79~0.98)	0.01
Median-stable group		0.63 (0.57~0.70)	<0.01		0.70 (0.61~0.81)	<0.01		0.69 (0.61~0.79)	<0.01
High-stable group		0.52 (0.45~0.59)	<0.01		0.60 (0.49~0.72)	<0.01		0.59 (0.50~0.71)	<0.01

The proximal LE8 score was the LE8 score from the examination at or before the cIMT measurement.

a Adjusted for age, sex, current drinker, education status, income status, and marital status.

b Adjusted for age, sex, current drinker, education status, income status, marital status, and baseline LE8 score.

c Adjusted for age, sex, current drinker, education status, income status, marital status, and proximal LE8 score.

### Sensitivity analysis

**Supplementary Tables S5**, **S6** (**Additional file 1**) show the results of sensitivity analysis. The baseline quintiles of LE8 score were significantly associated with continuous cIMT/high cIMT among male and female participants, participants without hypertension, participants without diabetes, participants without lipid-lowering drugs/antihypertensive drugs/hypoglycemic drugs, and participants in junior high school or below. We found similar results in the analysis of the association between LE8 score trajectories with continuous cIMT/high cIMT, except for female participants.

## Discussion

A significant finding of this study was that a high baseline LE8 score and keeping a high LE8 score were associated with lower continuous cIMT and attenuated risk of high cIMT in the study. Furthermore, the association between LE8 score trajectories and continuous cIMT/risk of high cIMT was found to be independent of baseline or proximal LE8 scores.

Since LS7 was proposed in 2010, the LS7 score has been widely used to measure CVH, and numerous studies have found that it is associated with decreased risk of cIMT, hypertension, CVD, and other related conditions. At present, there remains a lack of studies that have looked into the association between LE8 score and continuous cIMT/high cIMT since the new conception was first introduced. It is worth noting that our findings measured by new LE8 metrics were consistent with the results measured by the old LS7 metrics. The H3Africa AWI-Gen study, including 9,011 apparently healthy sub-Saharan African populations with a mean age of 51 years, demonstrated that poor CVH was associated with subclinical atherosclerosis measured by cIMT (25). Another study, consisting of 1,285 adults with a mean age of 29.7 years, found that ideal CVH metrics were related to positive CVH and a higher CVH score was associated with decreased cIMT (8). While there is a strong correlation between LS7 and LE8, LE8 has redefined and updated the components of LS7 to better address individual differences and changes. Due to the role of sleep health in cardiometabolic health and health outcomes, sleep health was added as a new component of LE8 (26, 27).

Furthermore, since the components of the LE8 metrics dynamic varied with time and environment, relying solely on the observation of the LE8 score has its limitations. Utilizing LE8 score trajectories to describe longitudinal changes can provide a more stable outcome. Our findings demonstrated that higher quartile groups of LE8 score trajectories led to a lower risk of high cIMT or continuous cIMT. This association was found to be independent of the baseline or proximal LE8 score. The previous studies about the association between LS7 trajectories and continuous cIMT/high cIMT were in keeping with this study’s results. A study including participants from five prospective cohorts indicated clinical CVH score trajectories were significantly associated with the level of subclinical atherosclerosis in middle adults, and independent of CVH at baseline and the time of cIMT measurement (28). Therefore, it is crucial for us to utilize appropriate preventive measures to maintain the long-term ideal status of each component of CVH to prevent and delay the progression of cIMT. By implementing lifestyle intervention and preventing risk factors, CVD could be prevented.

In a sensitivity analysis including non-hypertensive participants, a diminished reduction in cIMT was observed in participants with high baseline LE8 scores and LE8 score trajectories. Therefore, it is essential to maintain other components of LE8 metrics at an ideal level, even if blood pressure levels are well maintained. Additionally, we found that the association between the LE8 score and cIMT was stronger in participants in junior high school or below. The results suggest that participants with lower education levels may have poorer lifestyles, resulting in lower LE8 scores than those with higher education levels (29). This implies that individuals with low education levels could potentially benefit more from maintaining great CVH than those with higher education.

There are mounting studies showing that cumulative risk factors are associated with the risk of CVD. Even if blood pressure is controlled within the normal range, individuals with taking antihypertensive drugs have a higher risk of CVD compared with those not taking antihypertensive drugs. It is significant for individuals with risk factors to be treated; aggressively intervening in the progression of CVD risk factors may be an effective measurement to reduce the burden of CVD. Individuals could maintain an ideal CVH through timely and effective interventions and prevent incidents and the development of CVD. This is also supported by our study.

The strengths of the present study included the assessment of CVH using updated and enhanced LE8 metrics and the collection of multiple measurement data. Additionally, we described the longitudinal pattern of the LE8 score with trajectories and represented changes trends in the LE8 score. Nevertheless, there are certain limitations in this study. First of all, this study is a cross-sectional study, which cannot verify causality. Secondly, we did not observe the cIMT progression because we used only one data from the ultrasound scan. Thirdly, the participants in our study were all from the Kailuan study, and the findings have not been validated in other populations. Finally, taking drugs, education differences, hypertension, and diabetes may have some impact on results, but consistent results were obtained by sensitivity analysis.

## Conclusions

Our findings from our study suggested that the great CVH defined by the new LE8 metric at baseline and trajectories is associated with lower continuous cIMT and an attenuated risk of high cIMT. These findings suggested early aggressive intervention and maintaining high levels of CVH may be helpful in lowering cIMT.

## Data availability statement

The raw data supporting the conclusions of this article will be made available by the authors, without undue reservation.

## Ethics statement

This study was approved by the Ethics Committee of Kailuan General Hospital. All participants signed an informed consent form.

## Author contributions

QL, HC, SW, and WH designed this research. QL and HC conducted research. SC and DZ provided and monitored databases. QL, HC, and SC performed the statistical analysis. QL and HC wrote the manuscript. QL, HC, SC, DZ, SW, and WH made critical revisions to the manuscript. SW and WH had primary responsibility for the final content. All authors contributed to the article and approved the submitted version.
