# Supplementary material for: Association of baseline Life’s Essential 8 score and trajectories with carotid intima-media thickness
Source: Front Endocrinol (Lausanne). 2023 Jun 2;14:1186880. doi: 10.3389/fendo.2023.1186880 (PMC10272710; doi:10.3389/fendo.2023.1186880)
Supplement: Supplementary file 1 [file Table_1.docx]

***Supplemental Material***

**Association of baseline Life’s Essential 8 score and trajectories with carotid intima-media thickness**

**Qian Liu^1,^****^2^**^†^**, Haozhe Cui^3^**^†^**, Shuohua Chen^2^, Dongyan Zhang^4^, Wei Huang^4^**^*^**, Shouling Wu ^2^**^*^

**Table S1**. Definition and scoring approach for quantifying cardiovascular health in the Kailuan study.

**Table S2.** Age and sex specific cut point for defining high cIMT.

**Table S3** Baseline characteristics of participants according to sex in 2006.

**Table S4**. Baseline characteristics of participants by LE8 score trajectory groups.

**Table S5.** Association between baseline LE8 score and continuous cIMT/high cIMT in sensitivity analysis.

**Table S6.** Association between LE8 score trajectory groups and continuous cIMT/high cIMT in sensitivity analysis.

**Table S1. Definition and scoring approach for quantifying cardiovascular health in the Kailuan study.**

| Domain | Metric | Method of measurement | Quantification of CVH metric |
| --- | --- | --- | --- |
| Health behaviors | Diet health | Measurement: Self-reported intake of salt, fatty foods, and tea  Examples of salt intake measurement: “What flavor do you prefer.”  Examples of high fat food intake measurement: “How often do you eat fatty foods?”  Example of tea intake measurement: “How often do you drink tea?” | Metric: The unweighted average of salt, fatty food, and tea scoring.  Salt scoring:  Points Level  100 <6 g/day  50 6-12 g/day   1. >12 g/day   High fat food scoring:  Points Level  100 <1 time/week  50 1-3 times/week   1. >3 times/week   Tea scoring:  Points Level   1. ≥4 times/week   75 1-3 times/week  50 1-3 times/month  25 < 1 time/month  0 Never |
|  | Physical activity | Measurement: Self-reported times of physical activity (moderate or vigorous exercise) per week.  Example tools for measurement: “How many times did you usually spend on physical activity (note: It took at least 20 minutes each time)? ” | Metric: Minutes of physical activity per week.  Scoring:  Points Level  100 ≥ 60  50 20-60  0 <20 |
|  | Nicotine exposure | Measurement: Self-reported use of cigarettes  Example tools for measurement: Do you now smoke cigarettes? (Never smoker, former smoker, some days, every day) | Metric: Smoking status  Scoring:  Points Status  100 Never smoker  50 Former smokers quit ≥ 1 y  25 Current smokers,< 1cigarette/d  0 Current smoker, ≥1cigarette/d |
|  | Sleep health | Measurement: Self-reported average hours of sleep per night  Example tools for measurement: “On average, how many hours of sleep do you get per night?” | Metric: Average hours of sleep per night  Scoring:  Points Level  100 7 - <9 h  90 9 - < 10 h  70 6 - < 7 h  40 5 - <6 or ≥10 h  20 4 - <5 h  0 <4h |
| Health factors | BMI | Measurement: Body weight (kg) divided by height squared(m²)  Example tools for measurement: Objective measurement of height and weight | Metric: BMI (kg/m^2^)  Scoring:  Points Level  100 <23  75 23.0-24.9  50 25.0-29.9  25 30.0-34.9  0 ≥35.0 |
|  | Blood lipids | Measurement: Plasma TC and HDL-C with the calculation of non-HDL-C.  Example tools for measurement: Fasting blood sample.  non-HDL-C unit conversion:  1mmol/L= 38.67mg/L  1mg/L=0.02586mmol/L | Metric: Non-HDL-C (mmol/L)  Scoring:  Points Level  100 < 3.36  60 3.36-4.13  40 4.14-4.90  20 4.91-5.68  0 ≥ 5.69  If the drug-treated level subtracts 20 points |
|  | Blood glucose | Measurement: Fasting blood glucose(FBG)  Example tools for measurement: Fasting blood glucose sample.  HBA1C to FBG(mg/L) to conversion:  28.7 * A1C - 46.7 = FBG  FBG unit conversion:  1mg = 0.056mmol/L  1mmol/L= 18.02 mg/dL | Metric: FBG (mmol/L)  Scoring:  Points Level  100 No history of diabetes with FBG <5.6  60 No diabetes with FBG 5.6-6.9  40 Diabetes with FBG < 8.6  30 Diabetes with FBG 8.6-10.1  20 Diabetes with FBG 10.2-11.6  10 Diabetes with FBG 11.7-13.2  0 Diabetes with FBG ≥13.3 |
|  | Blood pressure | Measurement: Appropriately measured systolic and diastolic blood pressure  Example tools for measurement: Corrected Mercury sphygmomanometer | Metric: Systolic and diastolic blood pressure (mm Hg)  Scoring:  Points Level  100 <120 / < 80  75 120-129 / < 80  50 130-139 or 80-89  25 140-159 or 90-99  0 ≥160 or ≥100  Subtract 20 points if the treated level |

*Modified from American Heart Association’s New “Life’s Essential 8” Metrics. @Lloyd-Jones, D.M., et al., Life’s Essential 8: Updating and Enhancing the American Heart Association’s Construct of Cardiovascular Health: A Presidential Advisory From the American Heart Association. Circulation, 2022: p. 10.1161/CIR. 0000000000001078.

**Table S2. Age and sex specific cut points for defining high cIMT.**

|  | Sex | |
| --- | --- | --- |
| Age, years | Male | Female |
| <30 | 1.2 | 0.8 |
| 30~34 | 0.85 | 0.85 |
| 35~39 | 0.9 | 0.8 |
| 40~44 | 0.95 | 0.9 |
| 45~49 | 1.05 | 0.95 |
| 50~54 | 1.05 | 1 |
| 55~59 | 1.1 | 1 |
| 60~64 | 1.2 | 1.1 |
| 65-69 | 1.2 | 1.15 |
| 70~74 | 1.2 | 1.15 |
| 75~79 | 1.2 | 1.2 |
| 80~84 | 1.3 | 1.1 |
| 85~89 | 1.2 | 1.15 |
| ≥90 | 1.35 | 1.35 |

**Table 3 Baseline characteristics of participants according to sex in 2006.**

| Characteristic | Sex | | P value |
| --- | --- | --- | --- |
|  | Female | Male |  |
| Number of participants | 4777 | 8203 |  |
| Age, years | 45.16±11.53 | 50.30±11.27 | <0.01 |
| BMI, kg/m^2^ | 24.11±3.57 | 25.49±3.28 | <0.01 |
| Systolic blood pressure, mmHg | 120.52±19.85 | 134.31±19.27 | <0.01 |
| Diastolic blood pressure, mmHg | 77.62±10.68 | 86.08±11.51 | <0.01 |
| FBG mmol/L | 5.21±1.37 | 5.48±1.51 | <0.01 |
| HDL-C, mmol/L | 1.58±0.37 | 1.59±0.37 | 0.46 |
| TC, mmol/L | 4.83±1.16 | 4.75±1.39 | <0.01 |
| non-HDL-C, mmol/L | 3.25±1.13 | 3.17±1.10 | <0.01 |
| Sleep duration, hour | 7.54±2.78 | 7.27±2.09 | <0.01 |
| cIMT, mm | 0.81±0.21 | 0.92±0.39 | <0.01 |
| Hs-CRP (<3mg/L), n(%) | 4010(83.94) | 7093(86.47) | <0.01 |
| Marital status(In Married), n (%) | 4546(95.16) | 7981(97.29) | <0.01 |
| High school or above, n(%) | 1774(37.14) | 1468(17.90) | <0.01 |
| Income status (>1000RMB/month), n(%) | 465(9.73) | 566(6.90) | <0.01 |
| Current drinker, n(%) | 382(8.00) | 3527(43.00) | <0.01 |
| Smoking status, n(%) |  |  | <0.01 |
| Never | 4705(98.49) | 4550(55.47) |  |
| Former smokers quit ≥1 year | 15(0.31) | 526(6.41) |  |
| Current <1 cigarette/day | 15(0.31) | 293(3.57) |  |
| Current ≥1 cigarette/day | 42(0.88) | 2834(34.55) |  |
| Physical activity, n(%) |  |  | <0.01 |
| Never | 224(4.69) | 750(9.14) |  |
| 1-3 times/week | 3989(83.50) | 6200(75.58) |  |
| ≥4 times/week | 564(11.81) | 1253(15.27) |  |
| Sodium intake, n(%) |  |  | <0.01 |
| <6 g/day | 461(9.65) | 726(8.85) |  |
| 6-10 g/day | 3984(83.40) | 6540(79.73) |  |
| >10 g/day | 332(6.95) | 937(11.42) |  |
| High fat food intake, n(%) |  |  | <0.01 |
| <1 time/week | 451(9.44) | 567(6.91) |  |
| 1-3 times/week | 4077(85.35) | 6861(83.64) |  |
| >3 times/week | 249(5.21) | 775(9.45) |  |
| Tea intake, n(%) |  |  | <0.01 |
| Never | 3792(79.38) | 6109(74.47) |  |
| < 1 time/month | 289(6.05) | 312(3.80) |  |
| 1-3 times/month | 282(5.90) | 488(5.95) |  |
| 1-3 times/week | 208(4.35) | 425(5.18) |  |
| ≥4 times/week | 206(4.31) | 869(10.59) |  |
| Lipid-lowering drugs, n(%) | 42(0.93) | 69(0.88) | 0.79 |
| Antihypertensive drugs, n(%) | 458(9.68) | 807(10.00) | 0.54 |
| Diabetes history, n(%) | 304(6.36) | 747(9.11) | <0.01 |

Abbreviation: LE8, Life’s Essential 8; BMI, body mass index; TC, total cholesterol; LDL-C, low density lipoprotein cholesterol; HDL-C, high density lipoprotein cholesterol; non-HDL-C, non-high density lipoprotein cholesterol; Hs-CRP, high sensitive C-reactive protein; FBG, fasting blood glucose; cIMT, carotid intima-media thickness.

**Table S4. Baseline characteristics of participants by LE8 score trajectory groups.**

| Characteristic | LE8 score trajectory groups | | | | P value |
| --- | --- | --- | --- | --- | --- |
|  | Very low-stable group | Low-stable group | Median-stable group | High-stable group |  |
| Number of participants | 662 | 2944 | 3249 | 1904 |  |
| Age, years | 47.87±9.28 | 49.87±10.48 | 48.85±11.84 | 42.09±11.37 | <0.01 |
| Male, n(%) | 634(95.77) | 2493(84.68) | 2114(65.07) | 406(21.33) | <0.01 |
| Body mass index, kg/m^2^ | 27.59±3.28 | 26.31±3.23 | 24.94±3.17 | 22.46±2.68 | <0.01 |
| Systolic blood pressure, mmHg | 142.77±19.29 | 136.65±18.96 | 129.21±18.57 | 112.31±13.42 | <0.01 |
| Diastolic blood pressure, mmHg | 91.67±11.94 | 87.29±11.19 | 82.79±10.52 | 73.61±8.34 | <0.01 |
| Fasting blood glucose, mmol/L | 6.54±2.34 | 5.63±1.70 | 5.23±1.14 | 4.92±0.67 | <0.01 |
| HDL-C, mmol/L | 1.59±0.39 | 1.57±0.39 | 1.60±0.37 | 1.55±0.35 | <0.01 |
| Total cholesterol, mmol/L | 5.55±1.38 | 4.93±1.42 | 4.70±1.24 | 4.54±0.89 | <0.01 |
| non-HDL-C, mmol/L | 3.96±1.35 | 3.36±1.43 | 3.10±1.24 | 2.98±0.85 | <0.01 |
| Sleep duration, hour | 6.69±1.38 | 7.23±2.82 | 7.46±2.73 | 7.58±0.81 | <0.01 |
| IMT, mm | 0.98±0.62 | 0.91±0.35 | 0.87±0.33 | 0.78±0.16 | <0.01 |
| C-reactive protein (<3mg/L), n(%) | 533(80.51) | 2465(83.73) | 2806(86.37) | 1717(90.23) | <0.01 |
| Marital status(In Married), n (%) | 644(97.28) | 2855(96.98) | 3154(97.08) | 1818(95.53) | 0.01 |
| High school or above, n(%) | 144(21.75) | 582(19.77) | 775(23.85) | 783(41.15) | <0.01 |
| Income status (>1000RMB/month), n(%) | 61(9.21) | 237(8.05) | 242(7.45) | 193(10.14) | <0.01 |
| Current drinker, n(%) | 454(68.58) | 1298(44.09) | 826(25.42) | 286(15.03) | <0.01 |
| Smoking status, n(%) |  |  |  |  | <0.01 |
| Never | 103(15.56) | 1506(51.15) | 2581(79.44) | 1833(96.32) |  |
| Former smokers quit ≥1 year | 27(4.08) | 166(5.64) | 153(4.71) | 35(1.84) |  |
| Current <1 cigarette/day | 26(3.93) | 112(3.80) | 83(2.55) | 8(0.42) |  |
| Current ≥1 cigarette/day | 506(76.44) | 1160(39.40) | 432(13.30) | 27(1.42) |  |
| Physical activity, n(%) |  |  |  |  | <0.01 |
| Never | 178(26.89) | 310(10.53) | 184(5.66) | 62(3.26) |  |
| 1-3 times/week | 423(63.90) | 2201(74.76) | 2553(78.58) | 1572(82.61) |  |
| ≥4 times/week | 61(9.21) | 433(14.71) | 512(15.76) | 269(14.14) |  |
| Sodium intake, n(%) |  |  |  |  | <0.01 |
| <6 g/day | 53(8.01) | 274(9.31) | 288(8.86) | 223(11.72) |  |
| 6-10 g/day | 445(67.22) | 2308(78.40) | 2695(82.95) | 1581(83.08) |  |
| >10 g/day | 164(24.77) | 362(12.30) | 266(8.19) | 99(5.20) |  |
| Fatty food intake, n(%) |  |  |  |  | <0.01 |
| <1 time/week | 46(6.95) | 251(8.53) | 259(9.97) | 197(10.35) |  |
| 1-3 times/week | 490(74.02) | 2380(80.84) | 2769(85.23) | 1613(84.76) |  |
| >3 times/week | 126(19.03) | 313(10.63) | 221(6.80) | 93(4.89) |  |
| Tea intake, n(%) |  |  |  |  | <0.01 |
| Never | 436(65.86) | 2180(74.05) | 2522(77.62) | 1435(75.41) |  |
| < 1 time/month | 35(5.29) | 121(4.11) | 130(4.00) | 128(6.73) |  |
| 1-3 times/month | 65(9.82) | 183(6.22) | 172(5.29) | 122(6.41) |  |
| 1-3 times/week | 45(6.80) | 152(5.16) | 152(4.68) | 96(5.04) |  |
| ≥4 times/week | 81(12.24) | 308(10.46) | 273(8.40) | 122(6.41) |  |
| Lipid-lowering drugs, n(%) | 15(2.54) | 33(1.20) | 27(0.87) | 2(0.11) | <0.01 |
| Antihypertensive drugs, n(%) | 151(23.82) | 425(14.79) | 255(7.93) | 21(1.11) | <0.01 |
| Diabetes history, n(%) | 181(27.34) | 361(12.26) | 169(5.20) | 19(1.00) | <0.01 |

**Table S5. Association between baseline LE8 score and continuous cIMT/high cIMT in sensitivity analysis.**

|  | cIMT, mm | |  | high cIMT | |
| --- | --- | --- | --- | --- | --- |
|  | β(95%CI) | P value |  | RR(95%CI) | P value |
| Male(n=8203) | | | | | |
| Q1 | Reference |  |  | Reference |  |
| Q2 | -0.02(-0.04~0.00) | 0.11 |  | 0.68(0.59~0.79) | <0.01 |
| Q3 | -0.06(-0.09~-0.03) | <0.01 |  | 0.56(0.47~0.67) | <0.01 |
| Q4 | -0.08(-0.10~-0.05) | <0.01 |  | 0.60(0.51~0.72) | <0.01 |
| Q5 | -0.10(-0.14~-0.07) | <0.01 |  | 0.44(0.34~0.58) | <0.01 |
| Female(n=4777) | | | | | |
| Q1 | Reference |  |  | Reference |  |
| Q2 | -0.02(-0.05~0.01) | 0.14 |  | 0.69(0.49~0.98) | 0.04 |
| Q3 | -0.02(-0.05~0.00) | 0.09 |  | 0.75(0.54~1.03) | 0.07 |
| Q4 | -0.04(-0.07~-0.01) | <0.01 |  | 0.70(0.52~0.95) | 0.02 |
| Q5 | -0.06(-0.08~-0.03) | <0.01 |  | 0.52(0.38~0.71) | <0.01 |
| Participants without hypertension(n=7351) | | | | | |
| Q1 | Reference |  |  | Reference |  |
| Q2 | -0.01(-0.03~0.01) | 0.37 |  | 0.74(0.56~0.97) | 0.02 |
| Q3 | -0.02(-0.04~-0.01) | 0.01 |  | 0.68(0.52~0.90) | <0.01 |
| Q4 | -0.02(-0.04~-0.01) | <0.01 |  | 0.79(0.62~1.01) | 0.07 |
| Q5 | -0.03(-0.05~-0.01) | <0.01 |  | 0.62(0.48~0.80) | <0.01 |
| Participants without diabetes(n=11929) | | | | | |
| Q1 | Reference |  |  | Reference |  |
| Q2 | -0.01(-0.03~0.01) | 0.25 |  | 0.73(0.62~0.85) | <0.01 |
| Q3 | -0.04(-0.06~-0.02) | <0.01 |  | 0.65(0.56~0.77) | <0.01 |
| Q4 | -0.06(-0.08~-0.04) | <0.01 |  | 0.68(0.59~0.79) | <0.01 |
| Q5 | -0.07(-0.09~-0.05) | <0.01 |  | 0.54(0.45~0.64) | <0.01 |
| Participants without lipid-lowering drugs/antihypertensive drugs/ hypoglycaemic drugs(n=11549) | | | | | |
| Q1 | Reference |  |  | Reference |  |
| Q2 | -0.03(-0.05~-0.01) | <0.01 |  | 0.68(0.58~0.79) | <0.01 |
| Q3 | -0.05(-0.07~-0.03) | <0.01 |  | 0.63(0.54~0.74) | <0.01 |
| Q4 | -0.07(-0.09~-0.05) | <0.01 |  | 0.63(0.54~0.74) | <0.01 |
| Q5 | -0.08(-0.10~-0.06) | <0.01 |  | 0.49(0.41~0.59) | <0.01 |
| Participants with junior high school or below(n=9738) | | | | | |
| Q1 | Reference |  |  | Reference |  |
| Q2 | -0.02(-0.04~0.00) | 0.06 |  | 0.71(0.60~0.82) | <0.01 |
| Q3 | -0.05(-0.07~-0.03) | <0.01 |  | 0.61(0.52~0.73) | <0.01 |
| Q4 | -0.07(-0.09~-0.05) | <0.01 |  | 0.63(0.54~0.74) | <0.01 |
| Q5 | -0.09(-0.11~-0.06) | <0.01 |  | 0.49(0.40~0.61) | <0.01 |

Adjusted for age, sex, current drinker, education status, income status, marital status.

**Table S6. Association between LE8 score trajectory groups and continuous cIMT/high cIMT in sensitivity analysis**

|  | cIMT, mm | |  | high cIMT | |
| --- | --- | --- | --- | --- | --- |
|  | β(95%CI) | P value |  | RR(95%CI) | P value |
| Male(n=5647) | | | | | |
| Very low-stable group | Reference |  |  | Reference |  |
| Low-stable group | -0.07(-0.11~-0.04) | <0.01 |  | 0.82(0.74~0.91) | <0.01 |
| Median-stable group | -0.11(-0.14~-0.07) | <0.01 |  | 0.61(0.54~0.68) | <0.01 |
| High-stable group | -0.17(-0.22~-0.12) | <0.01 |  | 0.43(0.35~0.54) | <0.01 |
| Female(n=3111) | | | | | |
| Very low-stable group | Reference |  |  | Reference |  |
| Low-stable group | -0.01(-0.07~0.06) | 0.87 |  | 1.13(0.68~1.88) | 0.63 |
| Median-stable group | -0.04(-0.10-0.02) | 0.22 |  | 0.87(0.53~1.43) | 0.57 |
| High-stable group | -0.06(-0.12~0.00) | 0.06 |  | 0.71(0.43~1.18) | 0.19 |
| Participants without hypertension(n=5010) | | | | | |
| Very low-stable group | Reference |  |  | Reference |  |
| Low-stable group | -0.02(-0.04~-0.01) | 0.17 |  | 0.91(0.72~1.15) | 0.41 |
| Median-stable group | -0.04(-0.07~-0.02) | 0.01 |  | 0.75(0.60~0.95) | 0.02 |
| High-stable group | -0.06(-0.09~-0.03) | <0.01 |  | 0.61(0.47~0.78) | <0.01 |
| Participants without diabetes(n=8028) | | | | | |
| Very low-stable group | Reference |  |  | Reference |  |
| Low-stable group | -0.05(-0.08~-0.02) | <0.01 |  | 0.85(0.75~0.96) | <0.01 |
| Median-stable group | -0.08(-0.12~-0.05) | <0.01 |  | 0.63(0.56~0.72) | <0.01 |
| High-stable group | -0.11(-0.14~-0.07) | <0.01 |  | 0.52(0.45~0.60) | <0.01 |
| Participants without lipid-lowering drugs/antihypertensive drugs/ hypoglycaemic drugs(n=7782) | | | | | |
| Very low-stable group | Reference |  |  | Reference |  |
| Low-stable group | -0.10(-0.13~-0.06) | <0.01 |  | 0.83(0.74~0.93) | <0.01 |
| Median-stable group | -0.13(-0.16~-0.09) | <0.01 |  | 0.64(0.56~0.72) | <0.01 |
| High-stable group | -0.15(-0.18~-0.11) | <0.01 |  | 0.51(0.44~0.59) | <0.01 |
| Participants with junior high school or below(n=6474) | | | | | |
| Very low-stable group | Reference |  |  | Reference |  |
| Low-stable group | -0.08(-0.11~-0.04) | <0.01 |  | 0.79(0.71~0.89) | <0.01 |
| Median-stable group | -0.11(-0.14~-0.07) | <0.01 |  | 0.60(0.53~0.68) | <0.01 |
| High-stable group | -0.14(-0.18~-0.10) | <0.01 |  | 0.46(0.39~0.54) | <0.01 |

Adjusted for age, sex, current drinker, education status, income status, marital status.
